# Supplementary material for: MicroRNA Profiling of the Inflammatory Response after Early and Late Asthmatic Reaction
Source: Int J Mol Sci. 2024 Jan 22;25(2):1356. doi: 10.3390/ijms25021356 (PMC10817008; doi:10.3390/ijms25021356)
Supplement: Supplementary file 1 [file ijms-25-01356-s001.zip › Table S1.pdf]

Table S1. Dysregulated miRNAs in EAR / no LAR compared to controls

miRNA	baseMean	log2FoldChange	lfcSE	stat	pvalue	padj
hsa_piR_020497	3.400	4.215	1.088	3.873	0.000	0.065
hsa-miR-4435	2.610	4.574	1.232	3.712	0.000	0.065
hsa-miR-4746-5p	2.019	4.692	1.291	3.635	0.000	0.065
hsa-miR-5100	67.387	0.813	0.231	3.515	0.000	0.065
hsa-miR-6849-3p	2.331	-3.455	0.987	-3.502	0.000	0.065
hsa-miR-708-5p	2.052	4.270	1.262	3.383	0.001	0.084
hsa-miR-3928-3p	2.564	3.730	1.132	3.295	0.001	0.094
hsa-miR-4728-3p	2.178	3.896	1.191	3.270	0.001	0.094
hsa-miR-5196-3p	2.493	-2.915	0.910	-3.205	0.001	0.094
hsa-miR-6798-3p	2.234	-3.059	0.955	-3.202	0.001	0.094
hsa-miR-6797-3p	2.531	-3.044	0.958	-3.178	0.001	0.094
hsa-miR-6766-3p	2.764	-2.673	0.857	-3.120	0.002	0.094
hsa-miR-6808-3p	2.408	3.684	1.185	3.109	0.002	0.094
hsa-miR-6729-3p	2.003	-3.132	1.011	-3.097	0.002	0.094
hsa_piR_001170	30.446	0.923	0.299	3.087	0.002	0.094
hsa-miR-320a	3774.436	0.649	0.217	2.991	0.003	0.114
hsa-miR-4707-5p	1.549	3.821	1.283	2.978	0.003	0.114
hsa-miR-6069	7.240	1.246	0.419	2.975	0.003	0.114
hsa_piR_000765	121.761	0.601	0.205	2.937	0.003	0.122
hsa-miR-4487	1.489	-3.459	1.184	-2.921	0.003	0.122
hsa-miR-4664-5p	30.068	1.088	0.376	2.893	0.004	0.128
hsa-miR-320b	146.991	0.787	0.280	2.808	0.005	0.159
hsa-miR-493-5p	2.265	-2.557	0.921	-2.776	0.005	0.161
hsa-miR-1236-3p	3.545	-2.360	0.851	-2.773	0.006	0.161
hsa-miR-4781-5p	1.848	3.696	1.338	2.762	0.006	0.161
hsa-miR-1255a	46.974	-0.843	0.311	-2.713	0.007	0.180
hsa-miR-4433b-5p	103.805	-1.043	0.392	-2.661	0.008	0.202
hsa-miR-4433b-3p	1.578	4.349	1.645	2.643	0.008	0.203
hsa-miR-6825-3p	1.475	3.771	1.436	2.627	0.009	0.203
hsa-miR-6846-3p	2.056	-2.390	0.911	-2.622	0.009	0.203
hsa-miR-1908-5p	1.433	3.730	1.428	2.613	0.009	0.203
hsa-miR-4433a-3p	2.744	-2.395	0.941	-2.545	0.011	0.239
hsa-miR-6509-3p	3.686	3.843	1.522	2.525	0.012	0.246
hsa-miR-106a-5p	23.382	0.811	0.323	2.512	0.012	0.248
hsa-miR-330-3p	1.952	3.267	1.308	2.498	0.012	0.250
hsa-miR-1224-3p	2.702	-2.344	0.949	-2.470	0.014	0.262
hsa-miR-935	1.515	3.855	1.575	2.447	0.014	0.262
hsa-miR-548l	3.380	-2.096	0.857	-2.445	0.015	0.262
hsa-miR-23c	5.826	1.098	0.449	2.443	0.015	0.262
hsa_piR_019914	25.352	0.639	0.265	2.414	0.016	0.276
hsa-miR-4510	20.418	1.336	0.560	2.388	0.017	0.279
hsa-miR-4772-3p	22.258	0.984	0.413	2.384	0.017	0.279

hsa-miR-1908-3p	1.964	-2.387	1.001	-2.384	0.017	0.279
hsa-miR-500b-5p	52.313	0.522	0.222	2.346	0.019	0.296
hsa_piR_016735	114.008	0.597	0.255	2.345	0.019	0.296
hsa-miR-6734-5p	1.816	-2.625	1.124	-2.335	0.020	0.297
hsa-miR-548at-3p	1.379	-2.611	1.122	-2.328	0.020	0.297
hsa_piR_020814	1.455	3.707	1.599	2.318	0.020	0.299
hsa-miR-4433a-5p	2.462	-2.444	1.062	-2.301	0.021	0.306
hsa-miR-139-3p	1.172	3.889	1.733	2.244	0.025	0.341
hsa-miR-4732-5p	133.829	0.705	0.315	2.241	0.025	0.341
hsa-miR-589-5p	160.180	0.401	0.180	2.234	0.025	0.341
hsa-miR-2277-3p	51.364	0.583	0.261	2.229	0.026	0.341
hsa-miR-1292-5p	52.019	0.626	0.284	2.209	0.027	0.353
hsa_piR_001311	5.596	0.913	0.416	2.195	0.028	0.359
hsa-miR-6820-3p	9.473	-0.804	0.369	-2.182	0.029	0.360
hsa-miR-1272	32.564	0.680	0.313	2.173	0.030	0.360
hsa_piR_001312	28.418	0.566	0.262	2.162	0.031	0.360
hsa_piR_018570	94.236	0.455	0.210	2.162	0.031	0.360
hsa-miR-6859-5p	3.120	-1.786	0.827	-2.160	0.031	0.360
hsa-miR-25-3p	94412.794	0.415	0.194	2.137	0.033	0.374
hsa-miR-103a-2-5p	21.476	0.967	0.454	2.128	0.033	0.377
hsa-miR-146b-3p	14.566	0.809	0.384	2.106	0.035	0.391
hsa-miR-30c-5p	17172.879	0.406	0.194	2.094	0.036	0.397
hsa-miR-337-3p	1.306	3.133	1.545	2.028	0.043	0.451
hsa-miR-5684	2.385	2.543	1.256	2.024	0.043	0.451
hsa-miR-942-3p	94.387	0.571	0.282	2.023	0.043	0.451
hsa-miR-3140-3p	3.936	-1.685	0.837	-2.014	0.044	0.454
hsa-miR-1255b-5p	35.087	0.469	0.236	1.988	0.047	0.468
hsa-miR-6882-3p	11.121	-0.790	0.398	-1.983	0.047	0.468
hsa_piR_020813	26.267	0.704	0.355	1.983	0.047	0.468
hsa-miR-3179	2.195	2.473	1.254	1.973	0.049	0.472
