# Supplementary material for: MicroRNA Profiling of the Inflammatory Response after Early and Late Asthmatic Reaction
Source: Int J Mol Sci. 2024 Jan 22;25(2):1356. doi: 10.3390/ijms25021356 (PMC10817008; doi:10.3390/ijms25021356)
Supplement: Supplementary file 1 [file ijms-25-01356-s001.zip › Table S2.pdf]

Table S2. Dysregulated miRNAs in EAR plus LAR compared to controls

miRNA	baseMean	log2FoldChange	lfcSE	stat	pvalue	padj
hsa-miR-4433b-3p	2.6522	4.9556	1.1037	4.4900	0.0000	0.0050
hsa-miR-493-5p	2.3422	-3.8703	0.9854	-3.9276	0.0001	0.0300
hsa_piR_020497	3.1430	3.9896	1.0479	3.8071	0.0001	0.0300
hsa-miR-15a-5p	3771.6453	-0.9822	0.2613	-3.7585	0.0002	0.0300
hsa-miR-374a-5p	355.4327	-1.2670	0.3431	-3.6932	0.0002	0.0310
hsa-miR-320a	4176.8315	0.6839	0.1904	3.5914	0.0003	0.0339
hsa_piR_020814	2.0874	4.1716	1.1731	3.5560	0.0004	0.0339
hsa-miR-1908-3p	2.0174	-3.6403	1.0343	-3.5196	0.0004	0.0339
hsa-miR-4435	1.8833	4.0247	1.1631	3.4604	0.0005	0.0339
hsa-miR-6509-3p	3.3541	3.6509	1.0578	3.4515	0.0006	0.0339
hsa-miR-32-5p	34.3247	-2.2247	0.6466	-3.4407	0.0006	0.0339
hsa-miR-1299	6.4711	1.8554	0.5410	3.4293	0.0006	0.0339
hsa-miR-4452	1.6067	4.2349	1.2385	3.4193	0.0006	0.0339
hsa-miR-15b-5p	18848.3267	-0.7914	0.2399	-3.2984	0.0010	0.0441
hsa-miR-548l	3.5371	-2.7883	0.8482	-3.2874	0.0010	0.0441
hsa-miR-6832-3p	1.7301	3.8909	1.1841	3.2859	0.0010	0.0441
hsa-miR-378c	188.4998	0.6400	0.1956	3.2719	0.0011	0.0441
hsa-miR-3928-3p	2.4765	3.6148	1.1118	3.2512	0.0011	0.0447
hsa-miR-146a-5p	998.1599	0.4914	0.1524	3.2245	0.0013	0.0462
hsa-miR-96-5p	583.9982	-0.8870	0.2765	-3.2075	0.0013	0.0462
hsa-miR-6797-3p	2.9691	-2.7621	0.8638	-3.1977	0.0014	0.0462
hsa-miR-20a-5p	1474.6951	-0.8323	0.2669	-3.1186	0.0018	0.0576
hsa-miR-6808-3p	3.7394	4.2503	1.3679	3.1072	0.0019	0.0576
hsa-miR-6766-3p	3.1786	-2.6100	0.8481	-3.0775	0.0021	0.0595
hsa-miR-139-3p	1.4411	4.0528	1.3194	3.0717	0.0021	0.0595
hsa-miR-4635	2.7896	-2.6577	0.8683	-3.0609	0.0022	0.0595
hsa-miR-424-5p	36.9837	-1.0937	0.3632	-3.0110	0.0026	0.0661
hsa-miR-3939	1.7679	-3.4377	1.1463	-2.9990	0.0027	0.0661
hsa-miR-708-5p	1.6037	3.8070	1.2707	2.9959	0.0027	0.0661
hsa-miR-337-3p	1.8379	3.5641	1.2150	2.9334	0.0034	0.0783
hsa-miR-3613-5p	122.8071	-1.1526	0.4046	-2.8483	0.0044	0.0942
hsa-miR-330-3p	2.0508	3.3111	1.1634	2.8460	0.0044	0.0942
hsa-miR-4677-5p	1.4470	3.6319	1.2763	2.8456	0.0044	0.0942
hsa-miR-548ay-5p	1.7619	3.4675	1.2235	2.8340	0.0046	0.0948
hsa-miR-4510	27.8496	1.7661	0.6254	2.8241	0.0047	0.0950
hsa-let-7i-5p	16371.5304	0.7234	0.2570	2.8150	0.0049	0.0950
hsa-miR-29b-3p	1598.3744	-0.6777	0.2418	-2.8026	0.0051	0.0961
hsa-miR-1304-3p	84.0172	0.7028	0.2614	2.6883	0.0072	0.1295
hsa-miR-320b	150.0537	0.6386	0.2376	2.6874	0.0072	0.1295
hsa-miR-3667-5p	18.5318	1.0273	0.3869	2.6550	0.0079	0.1390
hsa-miR-20b-5p	836.2309	-0.6308	0.2414	-2.6135	0.0090	0.1532
hsa-let-7b-5p	15201.0731	0.8486	0.3278	2.5889	0.0096	0.1581

hsa-miR-3179	2.7782	2.7876	1.0828	2.5744	0.0100	0.1581
hsa-miR-27a-3p	167.8322	-0.7084	0.2754	-2.5727	0.0101	0.1581
hsa-miR-3667-3p	7.7196	1.2932	0.5031	2.5707	0.0102	0.1581
hsa-miR-6729-3p	2.4307	-2.4106	0.9490	-2.5400	0.0111	0.1689
hsa-miR-29c-3p	960.2901	-0.5815	0.2333	-2.4926	0.0127	0.1775
hsa_piR_016742	1.5797	-2.8135	1.1313	-2.4869	0.0129	0.1775
hsa-miR-4433a-3p	3.1202	-2.3270	0.9357	-2.4868	0.0129	0.1775
hsa-miR-1236-3p	4.1638	-2.1031	0.8498	-2.4749	0.0133	0.1775
hsa-miR-1306-3p	10.5271	0.8997	0.3641	2.4706	0.0135	0.1775
hsa-miR-3143	9.2517	-0.8627	0.3504	-2.4617	0.0138	0.1775
hsa-miR-301a-3p	28.1463	-0.9083	0.3694	-2.4586	0.0139	0.1775
hsa-miR-5010-5p	3.5275	1.5881	0.6465	2.4565	0.0140	0.1775
hsa-miR-1285-3p	51.0851	-0.4679	0.1905	-2.4559	0.0141	0.1775
hsa-miR-3622a-3p	1.3404	-3.4229	1.3955	-2.4527	0.0142	0.1775
hsa-miR-6513-5p	3.8470	-2.1155	0.8663	-2.4420	0.0146	0.1796
hsa-miR-3960	1.3676	3.5253	1.4594	2.4156	0.0157	0.1898
hsa-miR-21-5p	2059.1753	-0.5953	0.2471	-2.4091	0.0160	0.1898
hsa-miR-4746-5p	1.3772	3.9998	1.6673	2.3990	0.0164	0.1898
hsa-miR-126-5p	634.5202	-0.8434	0.3518	-2.3973	0.0165	0.1898
hsa-miR-144-3p	251.3118	-1.4745	0.6173	-2.3888	0.0169	0.1911
hsa-miR-6849-3p	3.0197	-2.0642	0.8671	-2.3806	0.0173	0.1923
hsa-miR-4487	1.8445	-2.6026	1.0960	-2.3747	0.0176	0.1924
hsa-miR-4707-5p	1.3104	3.4674	1.4688	2.3608	0.0182	0.1967
hsa-miR-454-3p	563.6565	-0.5343	0.2278	-2.3456	0.0190	0.2012
hsa-miR-378a-3p	578.0606	0.4585	0.1958	2.3411	0.0192	0.2012
hsa-miR-20a-3p	8.8413	-0.8773	0.3806	-2.3048	0.0212	0.2157
hsa-miR-4714-3p	2.4188	-2.1238	0.9231	-2.3009	0.0214	0.2157
hsa-miR-18b-5p	1.8146	-2.5717	1.1189	-2.2985	0.0215	0.2157
hsa-miR-4433a-5p	2.7792	-2.3513	1.0388	-2.2635	0.0236	0.2318
hsa-miR-23c	6.7504	1.1683	0.5172	2.2588	0.0239	0.2318
hsa-miR-4676-3p	1.3726	-2.5616	1.1360	-2.2549	0.0241	0.2318
hsa-miR-4662a-5p	2.4994	-2.4239	1.0861	-2.2318	0.0256	0.2427
hsa-miR-27b-3p	178.7583	-0.4852	0.2180	-2.2253	0.0261	0.2436
hsa-miR-320c	48.3094	0.5864	0.2659	2.2056	0.0274	0.2529
hsa_piR_020829	14.4264	-0.6694	0.3062	-2.1862	0.0288	0.2622
hsa-miR-1294	62.6215	0.5732	0.2631	2.1787	0.0294	0.2638
hsa-miR-122-5p	10.3894	1.0930	0.5044	2.1668	0.0302	0.2668
hsa-miR-98-3p	2.3968	-2.1025	0.9715	-2.1642	0.0305	0.2668
hsa-miR-4732-5p	136.4168	0.5588	0.2597	2.1519	0.0314	0.2673
hsa-let-7e-5p	42.0489	0.5545	0.2580	2.1489	0.0316	0.2673
hsa-miR-140-5p	50.7629	-0.5594	0.2603	-2.1488	0.0316	0.2673
hsa-miR-1224-3p	3.2196	-1.9737	0.9287	-2.1251	0.0336	0.2802
hsa-miR-664a-5p	17.9793	0.6206	0.2936	2.1140	0.0345	0.2846
hsa-miR-194-5p	2852.2874	-0.3213	0.1523	-2.1093	0.0349	0.2847

hsa-miR-199b-3p	5.0360	-2.4295	1.1613	-2.0920	0.0364	0.2936
hsa-miR-26b-5p	3794.7920	-0.5571	0.2689	-2.0715	0.0383	0.3042
hsa-miR-4728-3p	1.1702	2.8249	1.3659	2.0681	0.0386	0.3042
hsa-miR-151a-3p	3333.9998	0.3935	0.1929	2.0396	0.0414	0.3224
hsa-miR-376a-3p	12.4015	-1.0534	0.5188	-2.0304	0.0423	0.3259
hsa-miR-144-5p	2013.2137	-0.7016	0.3474	-2.0197	0.0434	0.3280
hsa-miR-1273h-3p	11.9062	0.7120	0.3529	2.0174	0.0437	0.3280
hsa-miR-624-5p	20.3265	-0.6992	0.3471	-2.0143	0.0440	0.3280
hsa_piR_019420	1.4052	3.5792	1.7833	2.0070	0.0447	0.3287
hsa-miR-374a-3p	8.0803	-1.0412	0.5213	-1.9972	0.0458	0.3287
hsa-miR-589-5p	169.8919	0.3138	0.1574	1.9939	0.0462	0.3287
hsa-miR-4781-5p	1.1706	2.8413	1.4340	1.9814	0.0475	0.3287
hsa-miR-935	1.0727	3.1693	1.6005	1.9802	0.0477	0.3287
hsa-miR-142-5p	16319.9528	-0.3696	0.1869	-1.9780	0.0479	0.3287
hsa-miR-376c-3p	8.2364	-1.0672	0.5405	-1.9746	0.0483	0.3287
hsa-miR-6825-3p	1.0541	3.1277	1.5844	1.9740	0.0484	0.3287
hsa-miR-3688-5p	2.1648	-1.9179	0.9718	-1.9736	0.0484	0.3287
hsa-miR-548d-5p	14.2387	-0.6782	0.3442	-1.9706	0.0488	0.3287
