# Supplementary figures and images for: MicroRNA Profiling of the Inflammatory Response after Early and Late Asthmatic Reaction
Source: Int J Mol Sci. 2024 Jan 22;25(2):1356. doi: 10.3390/ijms25021356 (PMC10817008; doi:10.3390/ijms25021356)

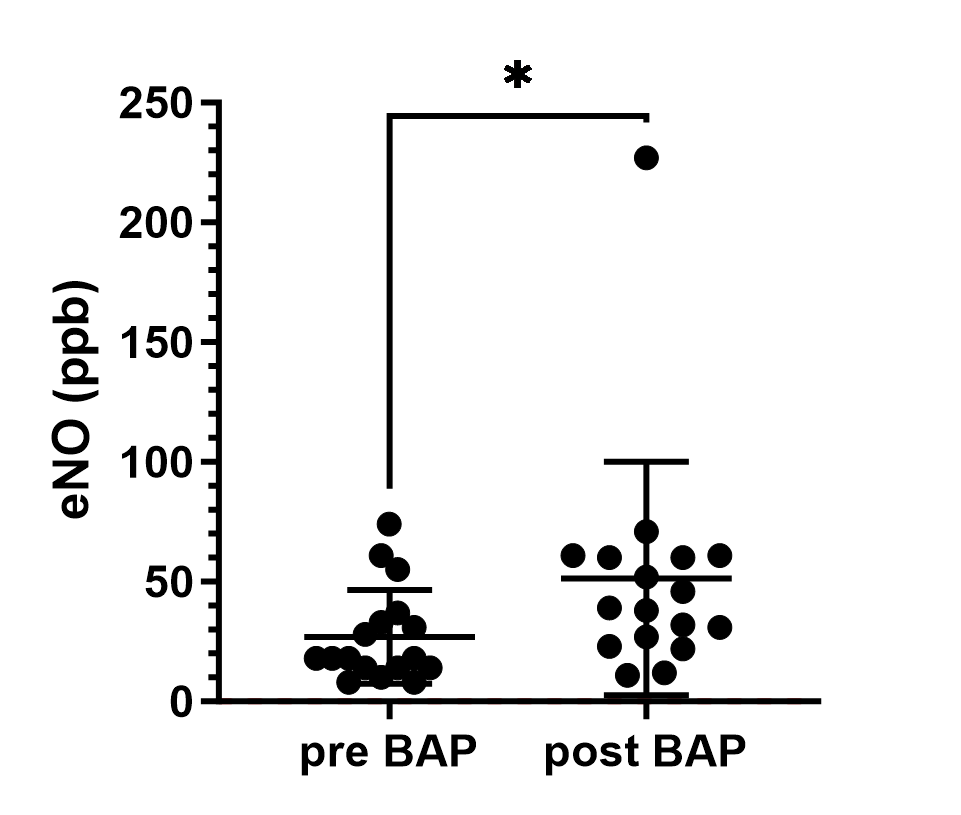

Supplement: Supplementary file 1 [file ijms-25-01356-s001.zip › Figure S1a.tif]

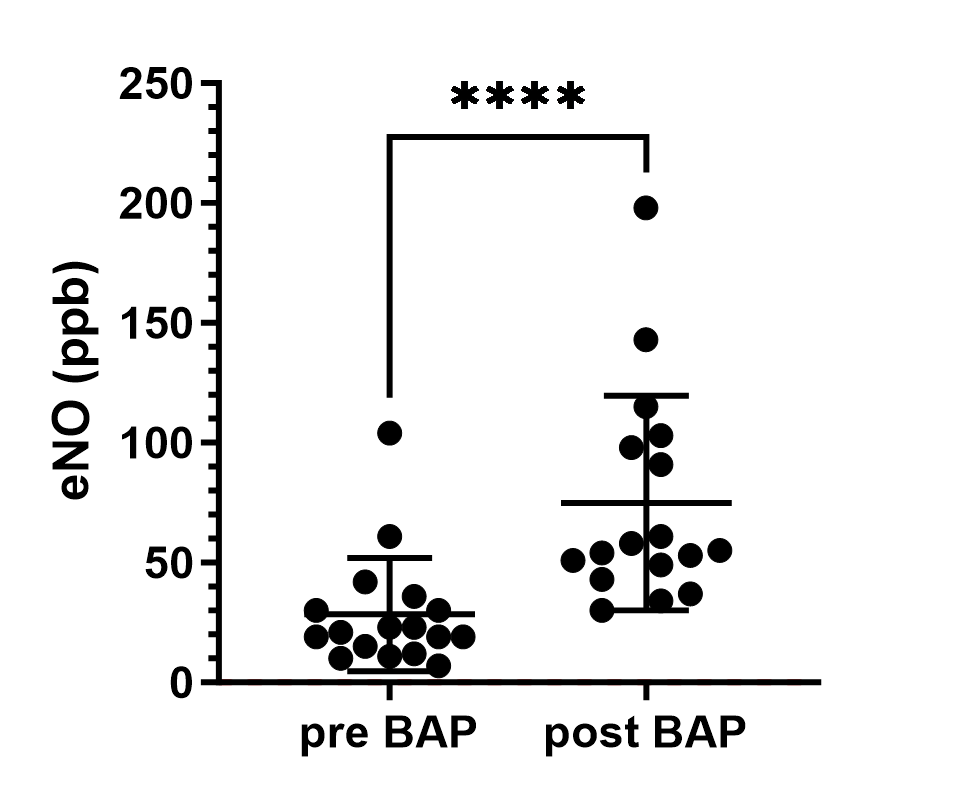

Supplement: Supplementary file 1 [file ijms-25-01356-s001.zip › Figure S1b.tif]

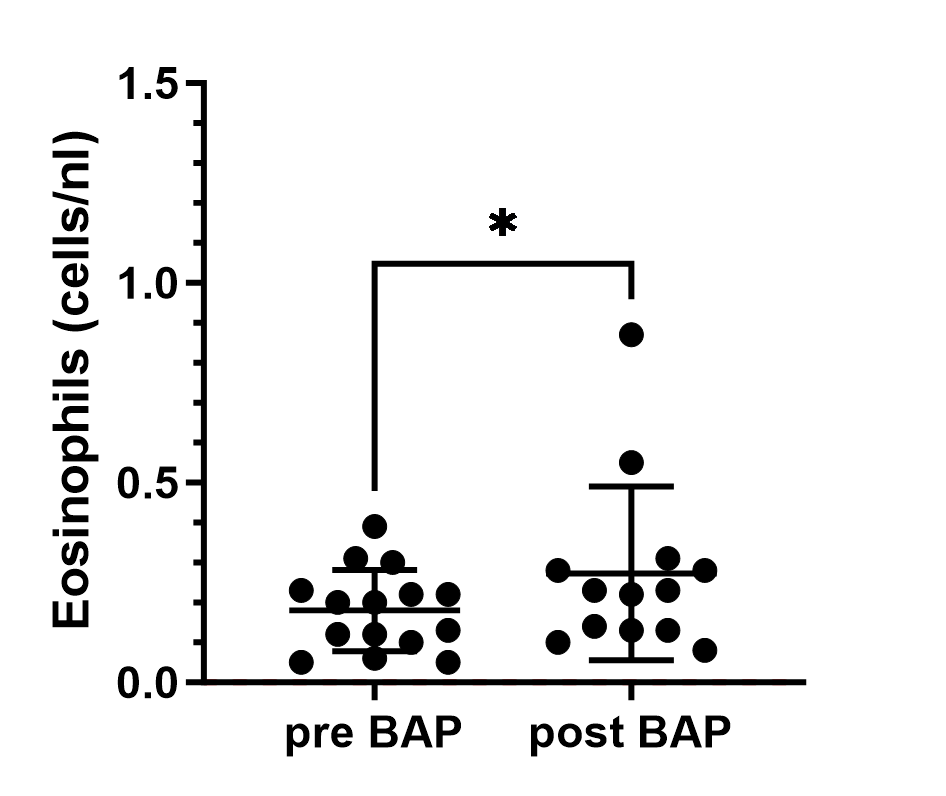

Supplement: Supplementary file 1 [file ijms-25-01356-s001.zip › Figure S1c.tif]

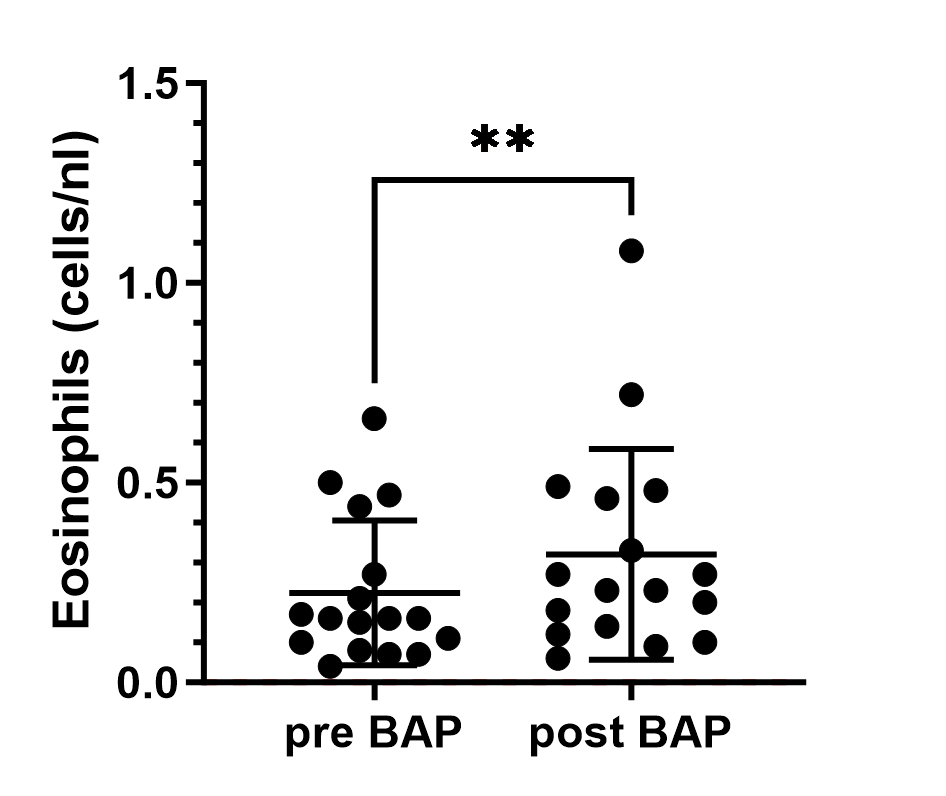

Supplement: Supplementary file 1 [file ijms-25-01356-s001.zip › Figure S1d.tif]

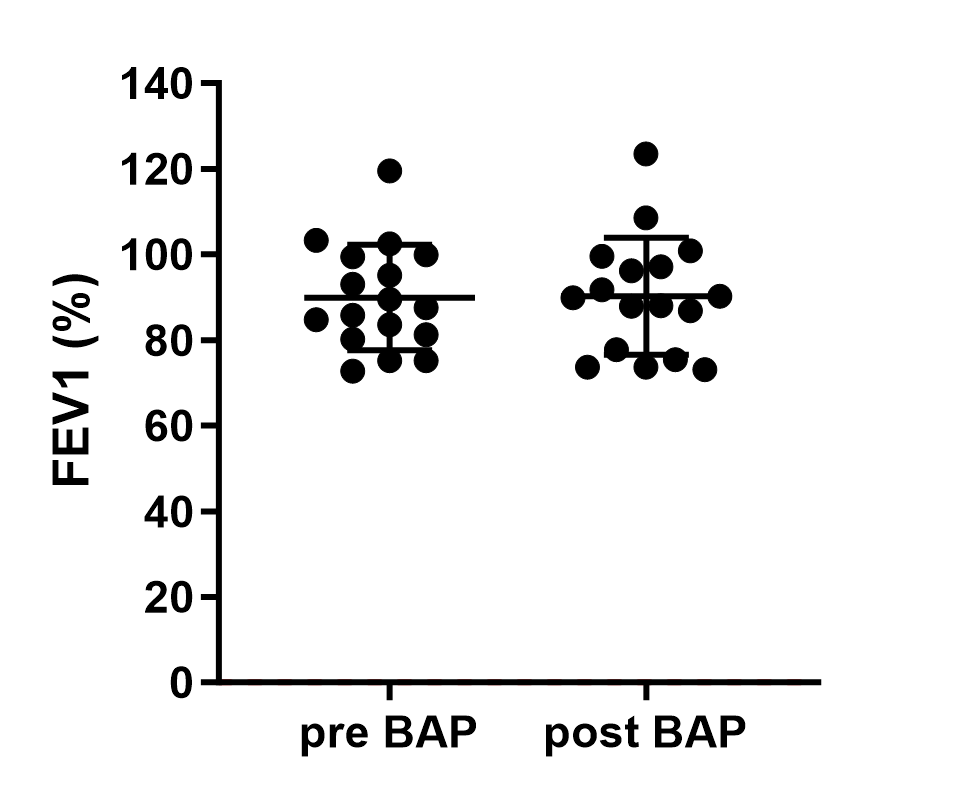

Supplement: Supplementary file 1 [file ijms-25-01356-s001.zip › Figure S1e.tif]

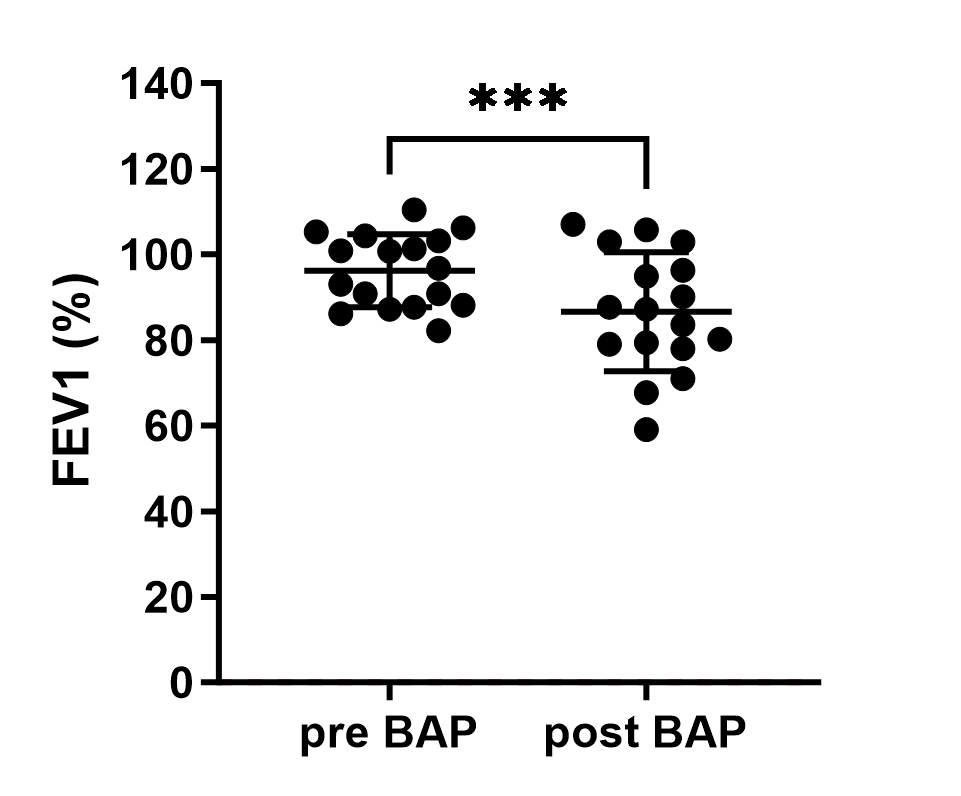

Supplement: Supplementary file 1 [file ijms-25-01356-s001.zip › Figure S1f.tif]

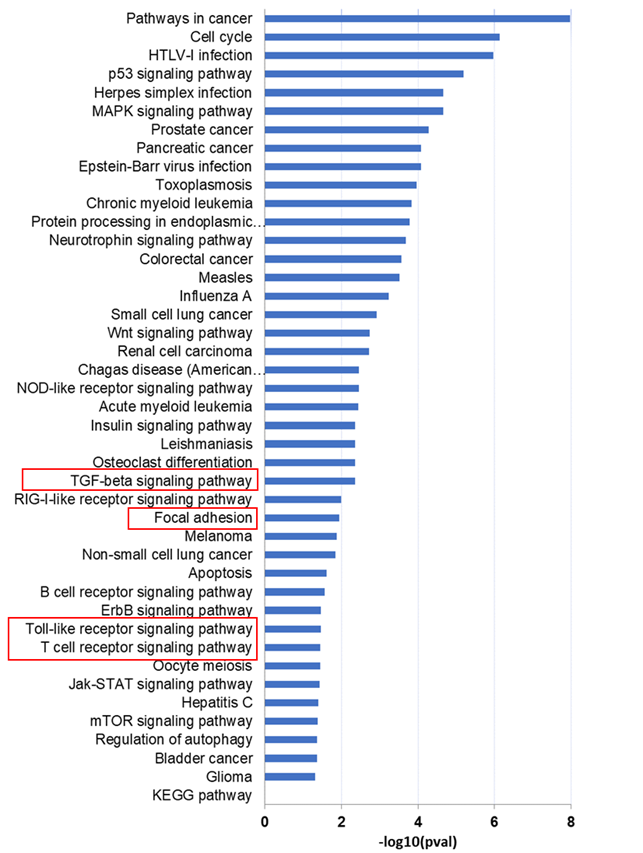

Supplement: Supplementary file 1 [file ijms-25-01356-s001.zip › Figure S2.tif]
